# Results of a feasibility cluster randomised controlled trial of a peer-led school-based intervention to increase the physical activity of adolescent girls (PLAN-A)

**DOI:** 10.1186/s12966-018-0682-4

**Published:** 2018-06-07

**Authors:** Simon J. Sebire, Russell Jago, Kathryn Banfield, Mark J. Edwards, Rona Campbell, Ruth Kipping, Peter S. Blair, Bryar Kadir, Kirsty Garfield, Joe Matthews, Ronan A. Lyons, William Hollingworth

**Affiliations:** 1 Centre for Exercise, Nutrition and Health Sciences, School for Policy Studies, University of Bristol, Bristol, UK; 2 Department of Population Health Sciences, Bristol Medical School, University of Bristol, Bristol, UK; 3 Centre for the Development and Evaluation of Complex Interventions for Public Health Improvement (DECIPHer), Bristol, UK; 4 Bristol Randomised Trials Collaboration, University of Bristol, Bristol, UK; 5 Farr Institute, Swansea University Medical School, Swansea, UK; 6 National Institute for Health Research (NIHR) Collaboration for Leadership in Applied Health Research and Care West (CLAHRC West) at University Hospitals Bristol NHS Foundation Trust, Bristol, UK

**Keywords:** Physical activity, Peers, Adolescent girls, Intervention, School

## Abstract

**Background:**

Most adolescent girls in the UK do not meet government physical activity recommendations and effective interventions are needed. This study reports the results of a feasibility trial of PLAN-A, a novel school-based peer-led physical activity intervention for adolescent girls.

**Methods:**

A two-arm cluster randomised controlled feasibility study was conducted in six English secondary schools (4 intervention & 2 control). Year 8 (age 12-13) girls were eligible and randomisation was at school-level. The intervention involved training Year 8 girls (out of school for two consecutive days, plus one top-up day 5 weeks later), who were identified by their peers as influential, to provide informal support to their friends to increase their physical activity. Feasibility of the intervention and the research was examined, including: recruitment, training attendance and data provision rates, evidence of promise of the intervention to affect weekday moderate-to-vigorous physical activity (MVPA), intervention cost and estimation of the sample size for a definitive trial. Accelerometer and questionnaire data were collected at the beginning of Year 8 (Time 0), the end of Year 8 (10-weeks after peer-supporter training) and the beginning of Year 9 (Time 2).

**Results:**

Four hundred twenty-seven girls were recruited (95% recruitment rate). 55 girls consented to be a peer-supporter and 53 peer-supporters were trained (97% of those invited). Accelerometer return rates exceeded 85% at each time point and wear time criteria was met by 83%, 71% and 62% participants at Time 0, 1 and 2 respectively. Questionnaire data were provided by >91% of participants at each time point. Complete-case adjusted linear regression analysis showed evidence of a 6.09 minute (95% CI = 1.43, 10.76) between-arms difference in weekday MVPA at Time 2 in favour of the intervention arm. On average PLAN-A cost £2685 per school to deliver (£37 per Year 8 girl). There were no adverse events. A trial involving 20 schools would be adequately powered to detect a between-arms difference in weekday MVPA of at least six minutes.

**Conclusions:**

The PLAN-A intervention adopts a novel peer-led approach, is feasible, and shows evidence of promise to positively affect girls’ physical activity levels. A definitive trial is warranted.

**Trial registration:**

ISCTRN, ISRCTN12543546, Registered on 28/7/2015, URL of registry record: http://www.isrctn.com/ISRCTN12543546

**Electronic supplementary material:**

The online version of this article (10.1186/s12966-018-0682-4) contains supplementary material, which is available to authorized users.

## Background

Physical activity is associated with benefits to young people’s physical and psychological health [1–3]. The UK and US governments recommend that children and adolescents undertake 60 minutes of moderate-to-vigorous physical activity (MVPA) each day [4, 5], however, many adolescents do not meet this guideline [6]. A consistent finding is that girls are less active than boys and that their physical activity declines at a faster rate than their male peers [7, 8]. Late childhood to mid adolescence is a key period of physical activity decline [9]. As such, there is a specific need to develop strategies to prevent the decline in, or increase adolescent girls’ physical activity.

Recent evidence syntheses have identified that previous interventions have had small positive effects on the physical activity of adolescent girls [10, 11], with one meta-analysis equating the average intervention effect to approximately 14% more physical activity in the intervention versus control group. Larger effects were shown for interventions targeting girls only, were multi-component, based in schools, utilised theory and targeted sedentary behaviour as well as physical activity [10, 11]. A recent trial in Australia of a whole-school, 24 month, multi-component intervention (Physical Activity 4 Everyone) resulted in a 7-minute (95% CI = 2.7, 11.4, p=0.002) increase in adolescents’ accelerometer-assessed MVPA compared to controls [12]. However, the intervention was less effective for girls (+4 minutes) than boys (+10 minutes).

Correlates of girls’ physical activity include physical self-perceptions, self-efficacy, perceived competence and enjoyment [13]. Yet, girls’ relationship with physical activity is also described as complex, including being intertwined with gender roles, societal norms and self-presentation [14]. Peer-influence, which increases as girls transition from childhood to adolescence [15], plays a role in girls’ physical activity [16, 17] and can comprise social support, presence of peers during physical activity, peer norms, friendship quality, changes to friendship groups, activity preferences of peers, affiliation to peer groups, and peer victimisation [17]. Despite being a key part of the complexity of girls’ physical activity, until recently peers have been relatively overlooked in many physical activity interventions [18]. Tymms et al. [19] reported results of a cluster randomised controlled trial of “MOVE” in England. MOVE was a cross-age peer-mentoring intervention in which Year 9 (age 13-14) students were trained to mentor same sex Year 7 students (age 11-12). The intervention comprised of six weekly 1:1 meetings (20-30 mins) aimed at identifying physical activity barriers and setting/reviewing goals using self-monitoring but there was no evidence that it affected (accelerometer-assessed) physical activity of the Year 7 students.

Despite the potentially important role of peers in adolescent girls’ physical activity, there is a lack of robust trials (Excluding Tymms et al. [19] and two ongoing studies [20, 21]) of peer-led physical activity interventions [22]. Previous interventions are limited to cross-age approaches, where older pupils support younger pupils (i.e. they are “peers” but not necessarily influential friends) using formal methods (e.g., mentoring or leading classes). A potential limitation of cross-age interventions is that pairs of pupils are unlikely to be friends or peers which may hamper the development of a foundation of trust, mutual understanding, shared experiences and key peer-support strategies (e.g., an older mentor may not be comfortable co-participating in physical activity with a younger pupil out of school). In the ASSIST (A Stop Smoking in Schools Trial) intervention [23] an alternative peer-led approach was used which involved students in a secondary school year group nominating influential peers who were trained as peer-supporters to informally diffuse messages and provide support to help their friends stop or not start smoking. In a cluster randomised controlled trial of 10,730 12-13 year olds from 59 schools in England and Wales the intervention reduced the odds of students being a smoker compared to controls (OR = 0.78, 95% CI = 0.64, 0.96) when data from three follow-ups was considered together.

Based on the success of ASSIST and the centrality of peers in young people’s physical activity, we hypothesised that a peer-diffusion intervention (i.e., informal peer-peer verbal support, encouragement, co-participation, sharing knowledge and shifting norms) that targeted adolescent girls’ physical activity could hold promise as a means of promoting physical activity. We therefore developed the PLAN-A intervention (Peer-Led physical Activity iNtervention) and in line with the UK Medical Research Council (MRC) framework on the development and evaluation of complex interventions [24], tested it in a feasibility trial [25, 26]. Specifically we aimed to assess recruitment and retention rates, estimate data provision and quality, estimate the potential effect of the intervention on accelerometer-derived week-day physical activity (evidence of promise), identify and collect the data needed to cost the intervention and estimate the sample size required to conduct a definitive trial.

## Methods

### Study Design

The study was a two-arm cluster randomised controlled feasibility study in six secondary schools to compare the PLAN-A intervention against a usual-practice control with embedded process and health economics evaluations. The study protocol was published in 2016 [25, 26] and was registered with ISCTRN (ISRCTN12543546) prior to data collection. A completed CONSORT checklist is in Additional file 1.

### Sampling and participants

**Fig. 1:**
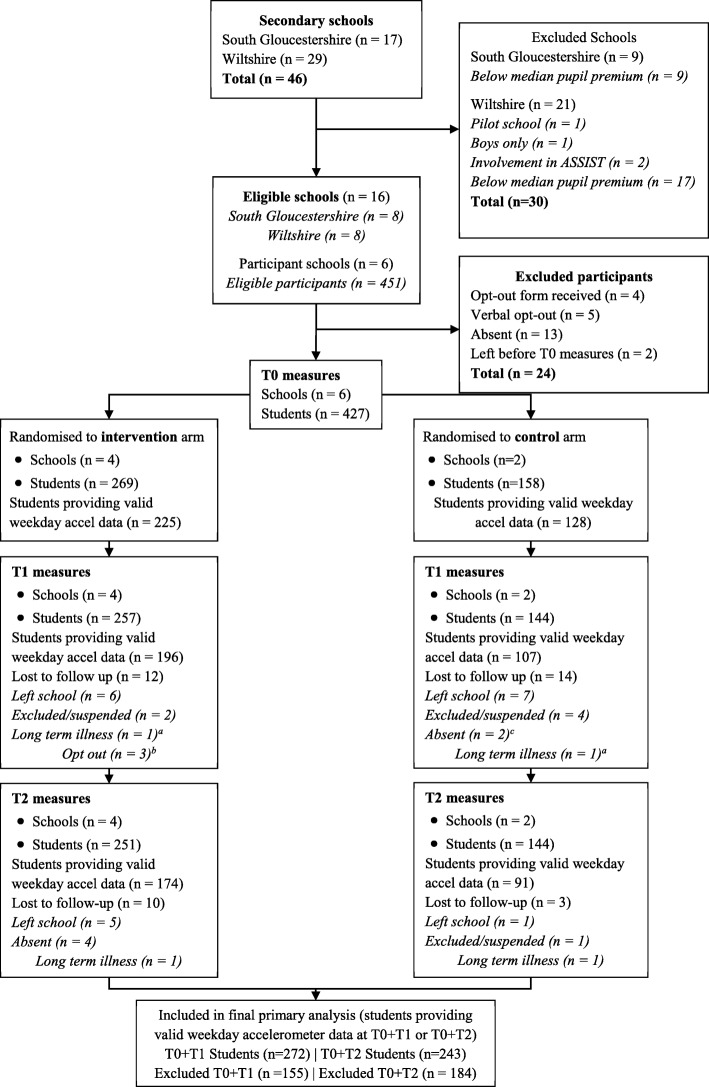
Trial profile for the PLAN-A study (based on CONSORT 2010 flow diagram). ^a^ Returned at T2, ^b^ Took part in T2, ^c^ Present at T2

Schools were eligible and invited to participate via letter and email to the Head Teacher if they were; state-maintained mainstream secondary schools in Wiltshire and South Gloucestershire (South West England), had girls in Year 8 (aged 12-13 years), were above the median of the local Pupil Premium Indicator (i.e., more deprived) [27], were not special educational needs schools and were not implementing ASSIST at the time. Since this was a feasibility study, a sample size calculation was not performed but we chose to recruit six schools as we expected this to provide sufficient variety of schools in which to test the feasibility of the research and the intervention. Of 46 secondary schools in the two areas, 30 were ineligible (26 were below median pupil premium) and the 16 remaining (eight in each area) were invited to participate and six were selected on a first-come-first served basis, in line with the stratification outlined below (Fig. 1). A face-to-face group recruitment pitch was made to Year 8 girls in each school and study information for students and parents was distributed. Ethical approval was obtained from the University of Bristol’s School for Policy Studies Research and Ethics committee (Ref: SPSREC14-15.A27). Parents could opt their child out of the study. Parents of peer-supporters provided written informed consent and peer-supporters gave written informed assent. All adults involved in the research (e.g., peer-supporter trainers) gave written informed consent. Pupils received a retail voucher (£5 for baseline & £10 for each follow up) in recognition of the time given to each data collection. Participating schools (intervention & control) received £500 and a summary of the findings in recognition of the time devoted to the study. An independent Trial Steering Committee provided study monitoring and oversight.

### Randomisation

Following the completion of baseline data collection, all schools were randomly allocated (school being the unit of randomisation), at an intervention:control ratio of 2:1 stratified within Local Authority area (Wiltshire & South Gloucestershire). Four schools were allocated to the intervention arm and two schools to the control arm by a member of the Bristol Randomised Trials Collaboration who was blind to the school identity and independent of the study. The statisticians, and all team members except the Project Manager, Research Associate and Fieldworkers were blind to allocation.

### Intervention description

The intervention was adapted from the ASSIST intervention through detailed formative research and its refinement and piloting is described in detail elsewhere [28]. Briefly, it comprised: (a) peer-supporter nomination, (b) a train-the-trainers programme, (c) peer-supporter training followed by a ten-week informal physical activity message diffusion period. Control schools did not receive the intervention but pupils completed identical measurements to the intervention schools. A TIDieR checklist is presented in Additional file 2.

#### Theoretical background

Commensurate with ASSIST, the PLAN-A intervention was primarily grounded in Diffusion of Innovations theory (DOI) [29] in which influential peers are identified as agents of change within a social system. In addition to DOI, the behaviour change principles and pedagogical approach within PLAN-A training were guided by self-determination theory (SDT) [30, 31] a framework which has been used previously to encourage the personal and social conditions needed to foster high quality (autonomous vs. controlled) motivation for physical activity amongst children and adolescents. Further explanation of SDT, and detailed examples of how it was incorporated into the train-the-trainers and peer-supporter training are reported elsewhere [25]. Briefly, trainers were educated about core SDT principles such as developing girls’ autonomous versus controlled motivation, and peer-supporter training tasks targeted developing participants’ autonomy, competence and/or relatedness and in turn using these principles to support their friends’ physical activity.

##### Train-the trainers programme

Peer-supporter training was delivered by female trainers who had attended a three-day (≈ 15 hours) education programme delivered by the study team (MJE, KB, JM & SS). Trainers were recruited as freelance individuals (e.g., youth workers, performing artists) or through physical activity/sport promotion teams to have a range of experiences in working with young people and physical activity promotion. Training covered gender inequalities in physical activity, the PLAN-A concept (informal diffusion) and theory in addition to teaching trainers about the sessions, activities and resources and giving time to practice delivery. Peer-supporter training was delivered by trainers working in pairs and trainers had time to work in their pairs and establish their delivery plan. Trainers were given a manual containing all resources needed.

##### Peer nomination

Prior to randomisation, Year 8 girls in all schools completed a four-item peer-nomination questionnaire to identify the influential girls in their school (i.e., *Who do you respect? Who are good leaders in sport or other group activities? Who do you trust? and Who do you look up to?*)*.* Girls could list up to five girls for each question. The 18% of girls with the most nominations were invited by letter to be a peer-supporter and attended a peer-supporter briefing where the role and training were explained and written information and consent forms were distributed. 18% were invited with the aim of recruiting 15% of the year group as peer-supporters in line with DOI.

##### Peer-supporter training

Consenting peer-supporters attended an initial two-day course, followed by a one-day top-up course approximately five weeks later. Separate training was held for each intervention school and took place off the school site (e.g., at a local business campus or community centre). Peer-supporters were provided with return transport from school to the training by coach (unless it was possible / more convenient to walk) and were chaperoned by a member of school staff. Training for one school was held on the school site due to the school being unable to release staff to chaperone. The interactive training blended education about the importance of physical activity, inspiration and empowerment of the girls to be active and development of the interpersonal skills needed to be a peer-supporter (See Table 1 in [25]). Peer-supporters were presented with many ideas of what types of support they could give (e.g., co-participation, encouraging and co-participating in incidental activity such as active travel to and from school, suggesting walking and talking at break/lunch instead of sitting, knowledge sharing, offering verbal support / being empathetic) as well as how to give support (e.g., how to be persuasive, language and style, and identifying who, where and when to support and not support). The top-up training revisited core learning objectives from the initial training, and focussed on sharing successes and collective problem solving. Peer-supporters each received a training booklet to support the training activities and a diary to record their peer-supporting if they wished (diaries were an intervention tool and data were not collected in). Following training, peer-supporters were asked to informally diffuse messages and norms about leading physically active lifestyles to their friends to encourage and support them to maintain or increase their physical activity.

**Table 1:** Participant and peer supporter recruitment rates by study school

School ID	Total N Year 8 girls	N (%) Opted-out	N (%) Did not participate (other reason)	N (%) consenting Year 8 girls	N (%^b^) invited to be peer-supporter	N (%^a^) consenting peer-supporters
Intervention arm						
2	58	0 (0.00)	3 (5.17)	55 (94.83)	13 (22.41)	13 (22.41)
3	96	3 (3.13)	5 (5.21)	88 (91.67)	18 (18.75)	17 (17.70) ^b^
4	66	0 (0.00)	2 (3.03)	64 (96.97)	12 (18.18)	11 (16.67)
6	69	0 (0.00)	7 (10.15)	62 (89.86)	14 (20.29)	14 (20.29)
Total	289	3 (1.04)	17 (5.88)	269 (93.07)	57 (19.72)	55 (19.03)
Control arm						
1	73	1 (1.37)	0 (0.00)	72 (98.63)	-	-
5	89	0 (0.00)	3 (3.37)	86 (96.63)	-	-
Total	162	1 (0.62)	3 (1.85)	158 (97.53)	-	-
School mean	75.16	0.67 (0.75)	3.33 (4.48)	71.16 (94.68)	14.25 (19.91)	13.75 (18.29)
Total	451	4 (0.89)	20 (4.43)	427 (94.68)	57 (19.72)	55 (19.03)

^a^ Percentage based on total N Year 8 girls in each intervention school

^b^ One nominated student attended the briefing but left the school the following day

### Data Collection

Data were collected at baseline (Time 0: the beginning of Year 8, September-October 2015), immediately post-intervention (Time 1: the end of Year 8, May-June 2016) and follow up (Time 2: the beginning of Year 9, September-October 2016).

#### Demographic information

At baseline (Time 0) participants reported their date of birth and ethnicity. Socio-economic position was estimated by: (1) an index of multiple deprivation (IMD) using home postcode and the English Indices of Deprivation [27] for each participant, (2) participant-reported receipt of free school meals and (3) the four-item family affluence scale [32].

#### Recruitment and retention to trial and intervention

Recruitment of schools and Year 8 girls (opt-out consent rate) was recorded at Time 0 and retention was recorded at Time 1 and 2. Recruitment of peer-supporters (consent rate) and peer-supporter trainers was recorded by the field team as well as attendance of peer-supporters at the briefing meeting and each peer-supporter training day.

#### Physical activity and sedentary time

Participants wore a waist-worn ActiGraph accelerometer (Model wGT3X-BT; ActiGraph LLC, FL, USA) during waking hours which recorded data at 30Hz for seven days. Participants were instructed to remove the accelerometer while sleeping, showering/bathing and participating in water sports. Scores derived from ActiGraph accelerometers have been shown to provide reproducible and valid estimates of physical activity amongst adolescents [33]. Periods of ≥ 60 minutes of zero counts were classified as non-wear and were removed. Accelerometer data provision was a primary outcome of the feasibility study, however for the purposes of assessing the secondary outcome of the potential effect of the intervention on girls’ MVPA, participants who provided at least two weekdays of at least 500 minutes of data between 05:00 and 23.59 were included in this analysis. The Evenson cut-point (≥2296 counts per minute)[34] was used to estimate mean daily minutes of MVPA on weekdays and weekend day(s) as this threshold is the most accurate for adolescents [35]. Participants’ weekday and weekend sedentary time was estimated using a cut point of ≤100 counts per minute [34].

#### Psychosocial Questionnaires

Full details of the psychosocial questionnaires will be published in a separate process evaluation paper. For the purposes of reporting data provision rates in this paper, pupils completed a survey (assessing physical activity motivation, psychological need satisfaction, self-esteem, physical activity-based self-efficacy, social support & peer norms) using Samsung Galaxy Table 4 tablet devices.

#### Economic analysis

The economic analysis aimed to assess the feasibility of collecting the data required to cost the intervention and conduct a cost-effectiveness analysis in a future definitive trial. The affordability and potential cost-effectiveness of the intervention was also explored. A public-sector perspective was taken assuming that if scaled up the intervention would be funded by schools or local government. Data were collected using expense claim and data collection forms, completed by the research team, students and school contacts. Students’ quality of life was assessed using the EQ-5D-Y [36]. Responses were mapped to a utility score (scores derived from adults were used in the absence of child specific scores [37]) at each time point and quality-adjusted life-years (QALYs) were estimated adjusting for baseline differences in utility scores [38].

### Data Analysis

Descriptive summary statistics were calculated by trial arm at baseline. Continuous data were analysed using means and standard deviations or median and inter-quartile range, where the data were markedly skewed. School and student recruitment and retention were presented as a CONSORT flow chart for schools by trial arm. Recruitment, retention and data provision were also summarised descriptively.

Summary statistics for the (definitive trial) primary and secondary outcomes are presented, by intervention and control group according to the allocation of the student’s school (i.e., an intention to treat (ITT) analysis). Mixed effects linear regression was used to estimate the adjusted differences in means between intervention and control groups. School-level variance in the outcome was accommodated by inclusion of school as a normal distribution random effect, and any remaining differences in the baseline assessment of the outcome measure, and local authority area (by which the randomisation was stratified), was accommodated by their inclusion as covariates. Each difference in means is presented with its 95% confidence interval. As this study was a feasibility study and not powered to detect differences between arms, p-values are not presented.

Two pre-specified sensitivity analyses were conducted assessing the impact of missing data. Firstly, where there were 5% or more missing MVPA measures, missing MVPA data were imputed for Time 1 and Time 2, in turn using Multiple Imputation by Chained Equations [39]. Fifty imputed data sets were generated for MVPA at each time point, and the mixed effects linear regression analysis described above was applied to these data. The variables used in the imputation equations have been reported elsewhere [40]. Secondly, MVPA measurements based on ≥ 1 valid day of accelerometer data (rather than ≥ 2 valid days) were used to estimate the adjusted difference between intervention and control groups in mean MVPA. The Trial Steering Committee also recommended an exploratory interaction analysis to examine whether any potential intervention effect was moderated by peer-supporter status. A binary covariate identifying peer-supporters and non-peer-supporters was added to the regression model for the analysis of MVPA, and p-value calculated for the test of the null hypothesis, of equal effect of the intervention on both groups.

The school-level intra-class correlation (ICC) coefficient for weekday MVPA was estimated in a variance component random effects analysis. As this gives an imprecise estimate in a study of this size, the data were compared with ICC estimates from other studies [12, 41, 42]. Sample sizes for a future definitive trial were calculated for assumed true differences in mean weekday MVPA between intervention and control groups of 6, 8 and 10 minutes, and for 80% and 90% statistical power at the two-sided 5% significance level. Sample size estimates were multiplied by the design effect: [1+(k-1)ICC], k being the number of participants at each school (cluster size = 70 informed by this study) and ICC the intra-cluster correlation coefficient for weekday MVPA. Sample size estimates were inflated to account for 30% loss to follow up on the primary outcome. All analyses were conducted in Stata (Version 14.2).

## Results

### School and participant recruitment

School and participant recruitment and retention are shown in Fig. 1 and Table 1. Within the six schools, 451 Year 8 girls attended the recruitment briefing. Four (0.89%) returned opt-out forms and 20 girls (4.43%) did not take part (no formal opt out) resulting in a 94.68% participation rate (N = 427 girls, intervention n =269; control n =158). Peer nomination resulted in 57 girls being invited to be a peer-supporter, 55 (96.49%) consented and 54 attended the training. The number of peer-supporters per school ranged from 11 (16.67% Year 8 girls) to 17 (17.71% Year 8 girls) (Table 1). Ten trainers with a range of experience in physical activity, youth work, or both, expressed an interest and five were recruited. All trainers were female (Mean age = 33.8, SD = 9.68, range = 21-45).

### Data provision

At each time point, accelerometer return rates exceeded 85% (Table 2). At Time 1 accelerometer return was approximately 10% lower in the control versus intervention arm. Compliance with the wear protocol was not different between arms at baseline, and was 5% and 7% lower in control arm at Times 1 and 2 respectively. At Times 0, 1 and 2, the wear time criteria was met by 82.63%, 71.13% and 62.21% of participants respectively. The psychosocial questionnaire was completed by >91% of participants at each time point and was similar between trial arms. The completion of the EQ-5D-Y exceeded 92% at each time point.

**Table 2:** Accelerometer and questionnaire data provision rates by trial arm at baseline, Time 1 and Time 2.

	Control, n (%)	Intervention, n (%^a^)	Total, n (% ^a^)
Time 0			
Accelerometer returned	153 (96.84)	258 (96.27)	411 (96.48)
Accelerometer valid^b^	128 (81.01)	224 (83.58)	352 (82.63)
Accelerometer invalid^c^	25 (15.82)	34 (12.69)	59 (13.85)
Accelerometer missing^d^	5 (3.16)	10 (3.73)	15 (3.52)
Psychosocial questionnaire	157 (99.37)	269 (100.00)	426 (99.77)
Time 1			
Accelerometer returned	135 (85.44)	255 (95.15)	390 (91.55)
Accelerometer valid	107 (67.72)	196 (73.13)	303 (71.13)
Accelerometer invalid	27 (17.09)	54 (20.15)	81 (19.01)
Accelerometer missing	24 (15.19)	18 (6.71)	42 (9.86)
Psychosocial questionnaire	144 (91.14)	257 (95.54)	401 (93.91)
Time 2			
Accelerometer returned	140 (88.61)	241 (89.93)	381 (89.44)
Accelerometer valid	91 (57.59)	174 (64.93)	265 (62.21)
Accelerometer invalid	44 (27.85)	66 (24.63)	110 (25.82)
Accelerometer missing	23 (14.56)	28 (10.45)	51 (11.97)
Psychosocial questionnaire	144 (91.14)	251 (93.31)	395 (92.51)
Time 0 and Time 1			
Valid accelerometer data	95 (60.13)	177 (66.04)	272 (63.85)
Psychosocial questionnaire	144 (91.14)	257 (95.54)	401 (93.91)
Time 0 and Time 2			
Valid accelerometer data	81 (51.27)	162 (60.45)	243 (57.04)
Psychosocial questionnaire	144 (91.14)	251 (93.31)	395 (92.51)

^a^ One participant excluded as they could not wear accelerometer: accelerometer results are presented as a % of 268 for Intervention total and 426 for overall total.

^b^ Accelerometer worn, wear criteria met (≥ 2 valid weekdays, defined as ≥ 500 minutes of data between 05:00 and 23.59).

^c^ Accelerometer worn, wear criteria not met.

^d^ No data provided

### Baseline data

The trial arms were well balanced at baseline (Table 3). IMD (i.e., deprivation) was slightly higher in the intervention versus control group although both medians were in the second quintile range (8.5 to 13.8) of least deprived households in England [27]. Weekday and weekend minutes of MVPA were similar between arms. Participants in the intervention group recorded more minutes of sedentary time on weekdays and overall. Peer-supporters had higher family affluence and similar IMD to non-peer-supporters and a greater proportion of peer-supporters than non-peer-supporters were of white ethnicity (Additional file 3). Peer-supporters recorded approximately 12 minutes more MVPA on weekdays and weekends and less sedentary time (mainly due to weekday activity) than non-peer-supporters. Physical activity guidelines were met by 59% of peer-supporters versus 35% of non-peer-supporters. Peer-supporters also reported higher EQ-5D-Y scores.

**Table 3:** Baseline (Time 0) descriptive data by trial arm.

	Control		Intervention	
Variable	n	Mean ± SD / Median (LQ, UQ) / %	n	Mean ± SD / Median (LQ, UQ) / %
Age	158	13.53 ± 0.29	269	13.50 ± 0.30
IMD	134	9.99 (7.09, 16.50)	245	11.93 (6.69, 18.60)
Family affluence	157	6.92 ± 1.70	269	6.96 ±1.59
Receiving free school meals (n, %)	22	14.01	30	11.15
Ethnicity – White British (n, %)	125	79.62	232	86.25
Ethnicity – White other (n, %)	9	5.73	9	3.35
Ethnicity – Mixed (n, %)	5	3.19	7	2.60
Ethnicity – Other (n, %)	18	11.47	21	7.81
Weekday MVPA (min)	128	56.38 (45.44, 73.60)	225	52.50 (40.67, 68.90)
Weekend MVPA (min)	69	38.58 (21.50, 62.00)	155	38.33 (23.83, 59.58)
Overall MVPA (min)	128	54.51 (42.98, 71.42)	225	50.50 (40.33, 63.83)
Weekday sedentary (min)	128	467.07 (418.03, 524.73)	225	505.63 (450.57, 551.00)
Weekend sedentary (min)	69	428.25 (367.25, 520.17)	154	443.96 (393.17, 513.58)
Overall sedentary (min)	128	387.25 (59.36, 487.71)	225	454.13 (75.70, 519.81)
60 mins MVPA per weekday (n, %)	54	42.19	87	38.67
60 mins MVPA per weekend day (n, %)	20	28.99	36	23.23
EQ-5D-Y	153	79.09 ± 16.91	260	72.00 ± 18.74

IMD = Index of Multiple Deprivation; MVPA = moderate-to-vigorous physical activity

### Attendance at peer-supporter training

Almost all (94%; (53/55)) peer-supporters attended all of the training (Table 4). In the majority of cases, absences were due to illness. Attendance at training was not different in the school where training was delivered on the school site (School 4), compared to the off-site training for pupils in the other schools. There were no adverse events.

**Table 4:** Attendance at the peer-supporter training

School ID (N Yr 8 girls)	Attendance Day 1 (N (%))	Attendance Day 2 (N (%))	Attendance days 1 and 2 (N (%))	Attendance at top-up (N (%))	Attendance at all 3 days (N (%))
2 (55)	13 (100.00)	13 (100.00)	13 (100.00)	12 (92.31)^d^	12 (92.31)
3 (88)	17 (100.00)	17 (100.00)^b^	17 (100.00)	17 (100.00)	17 (100.00)
4 (64)	10 (90.91)^a^	11 (100.00)	10 (90.91)	11 (100.00)	10 (90.91)
6 (69)	13 (92.86)^a^	13 (92.86)^a^	13 (92.86)	13 (92.86)^c^	13 (92.86)

All %s are based on the N of consenting peer-supporters (Table 1).

^a^Participant illness.

^b^Participant unwell in a.m., attended p.m.

^c^One participant did not attend either of the first two days of training due to illness so 13 of 14 girls were invited to attend top-up training.

^d^Parent did not want participant missing a day of school

### Evidence of Promise

There was no evidence of a difference in weekday or weekend MVPA between intervention and control groups at Time 1 (Table 5). There was some evidence that the intervention group were less sedentary on weekdays than the control group at Time 1 (-31.8 minutes, 95% CI = -57.44, -6.18). At Time 2, there was evidence that pupils in the intervention arm performed more weekday MVPA than controls (6.09 minutes, 95% CI = 1.43, 10.76) which reflected the intervention group maintaining their baseline level of MVPA while the MVPA of the control group participants decreased over time. Similar to Time 1, there was some evidence that the intervention group were less sedentary on weekdays at Time 2 (-23.26 minutes, 95% CI = -43.73, -2.79). The confidence intervals around the estimated differences in weekend sedentary behaviour were too wide to indicate a potential difference between groups. In both arms EQ-5D-Y scores decreased between Time 0 and Time 1 and increased between Time 1 and Time 2 (Additional file 4). Differences between groups for unadjusted EQ-5D-Y scores and estimated QALYs were small with confidence intervals including (or close to) zero. Sensitivity analysis based on the imputed data set and when the criterion for participant inclusion based on their accelerometer data was relaxed to ≥1 valid day were very similar to the complete case analysis in both point estimate and 95% confidence intervals (Additional file 5). There was some evidence that peer-supporters benefitted more from the intervention than non-peer supporters at Time 1, but no evidence of a difference between the groups at Time 2 (Additional file 6).

**Table 5:** Adjusted between-group differences in physical activity variables at Time 1 and Time 2

	Control		Intervention		
Variable	n	Mean ± SD	n	Mean ± SD	Intervention vs. control adjusted difference in means (95% CI)^a^
	Time 0				
Mean weekday MVPA (mins)^b^	128	59.79 ± 21.57	225	55.07 ± 21.57	-
Mean weekend day MVPA (mins)	69	45.33 ± 31.31	154	45.11 ± 30.53	-
Mean weekday sedentary (mins)	128	471.84 ± 73.98	225	503.40 ± 79.74	-
Mean weekend sedentary (mins)	69	442.05 ± 121.07	154	451.11 ± 95.00	-
	Time 1				
Mean weekday MVPA (mins)^b^	95	61.19 ± 22.10	177	60.72 ± 22.45	1.11 [-4.31, 6.55]
Mean weekend day MVPA (mins)	37	45.07 ± 30.21	91	41.80 ± 27.49	-3.88 [-13.57, 5.81]
Mean weekday sedentary (mins)	95	522.37 ± 87.74	177	509.87 ± 81.67	-31.8 [-57.44, -6.18]
Mean weekend sedentary (mins)	37	461.29 ± 112.19	91	534.73 ± 188.56	67.43 [-51.34, 186.20]
	Time 2				
Mean weekday MVPA (mins)^b^	81	54.31 ±19.56	162	58.65 ± 22.21	6.09 [1.43, 10.76]
Mean weekend day MVPA (mins)	35	37.95 ± 32.14	74	46.57 ± 37.52	11.45 [-2.25, 25.15]
Mean weekday sedentary (mins)	81	511.65 ± 103.21	162	510.78 ± 85.29	-23.26 [-43.73, -2.79]
Mean weekend sedentary (mins)	35	473.06 ± 93.74	74	471.95 ± 97.20	-5.25 [-41.19, 30.69]

^a^ The control group is the reference group for between group comparisons. Models are adjusted for baseline outcome value, N valid days accelerometer data & local authority and school-level clustering.

^b^ *A priori* primary outcome in a future definitive trial

### Sample Size

The intra-class correlation (ICC) on weekday minutes of MVPA (i.e., the primary outcome for a definitive trial) from the PLAN-A sample at each time point was; Time 0: <0.00 (95% CI = 0.0, 0.09), Time 1: 0.02 (95% CI = 0.0, 0.06) and Time 2: <0.0001 (95% CI = 0.0, 0.03). Based on these findings, and ICCs from other studies involving adolescent girls, sample size calculations for a definitive trial were based on an ICC of 0.01 (and were also modelled for scenarios where the ICC was 0.02 & 0.03). To detect a 10-minute between arms difference in weekday MVPA with 90% power, an alpha of 5%, and with a design effect of 1.7 (based on 70 students per school), 560 students are required (280 per arm) (Table 6). This was inflated to 800 students in 12 schools to account for 30 per cent loss to follow-up. Using a smaller between-arms difference in MVPA of 6 minutes (i.e., the point estimate from this feasibility study) and keeping all other factors constant, 980 students would be required (490 per arm), inflated to 1400 students (700 per arm) in 20 schools to account for 30% loss to follow-up.

**Table 6:** Sample size calculation for a future definitive trial

Between-arms difference in weekday MVPA (mins)	Power	N students (uninflated)	N students (inflated)	N Schools
10	90	560	800	12
10	80	420	600	10
8	90	700	1000	16
8	80	560	800	12
**6**	**90**	**980**	**1400**	**20**
6	80	840	1200	18

The following parameters were used: cluster size = 70, ICC on weekday MVPA = 0.01 MVPA standard deviation = 20 minutes, correlation between baseline and follow-up MVPA = 0.4, 5 per cent two-sided alpha and inflation to account for 30% of participants not providing primary outcome data.

Values in boldface represent the sample size values proposed for the primary outcome in a future definitive trial

### Costs

The average cost per school of delivering the two-day and top-up day peer-supporter training were £1490 and £794 respectively, and the total cost of delivering the intervention in each school ranged from £2309 to £3235 (average £2685 per school and £37 per Year 8 girl) (Table 7). Trainer time and travel costs, whether the students and school contact incurred travel costs and whether the training was delivered in or outside of school were key cost drivers. Peer-supporters who attended the student briefing, peer nomination, peer-supporter meeting and all three days of training spent on average 16 hours and 34 minutes taking part in PLAN-A.

**Table 7:** Costs of intervention delivery

Item	All schools		
	Total cost (£)	Average cost per school (£)	Average cost per Year 8 girl (£)
Pre-intervention preparation	490.55	122.64	1.70
Student briefing	223.71	55.93	0.77
Peer nomination	444.35	111.09	1.54
PS meeting	383.20	95.80	1.33
Two-day PS training			
Trainer fees and travel	2940.22	735.06	10.17
Student and school contact travel	440.00	110.00	1.52
Venue hire ^a^	566.80	141.70 (188.93)	1.96
Refreshments	904.40	226.10	3.13
Resources and equipment	1109.51	277.38	3.84
**Two-day PS training total**	**5960.93**	**1490.23**	**20.63**
Top-up PS training			
Trainer fees and travel	1427.26	356.82	4.94
Student and school contact travel	245.00	61.25	0.85
Venue hire ^a^	332.40	83.10 (110.80)	1.15
Refreshments	539.14	134.79	1.87
Resources and equipment	630.97	157.74	2.18
**Top-up PS training total**	**3174.77**	**793.69**	**10.99**
Post-intervention materials	63.84	15.96	0.22
**Total intervention cost**	**10741.35**	**2685.34**	**37.17**

^a^ Peer supporter training for one school was held on the school site and incurred no venue hire cost. Average venue hire cost was (£188.93 & 110.80 for the two-day and top up days training respectively) across the three schools

## Discussion

This study showed that the PLAN-A intervention can be delivered and that it is feasible to conduct the research required to evaluate its effectiveness and cost-effectiveness in a definitive trial. The study was acceptable to schools and students with approximately 95% of eligible students in the six study schools participating at baseline and 93% of these were retained at the final follow up. This level of involvement is important for the potential effectiveness of the intervention and evaluation of its effect given that PLAN-A operates at the whole school year level.

At baseline approximately 60% of girls did not meet the recommendations of 60 minutes of MVPA per day and average MVPA levels were approximately 50 and 40 minutes per week day and weekend day respectively. These findings are similar to a recent UK accelerometer study which showed that 68% of adolescent girls performed less than 60 minutes MVPA per day [43] and support the present and future research efforts to increase girls’ physical activity.

Recruitment of peer-supporters was successful as 97% of those nominated and invited consented to the role and 94% attended all the peer-supporter training, suggesting that the peer-supporter concept was acceptable to adolescent girls. Peer-supporters were more physically active on average than non-peer-supporters at baseline showing that more active girls had been nominated to be peer-supporters. However, 40% of peer-supporters did not meet the recommended 60 minutes of MVPA per day. Peer-supporter training was delivered as planned at a non-school venue for three of the four intervention schools and in one school was delivered on the school site as financial/staffing constraints prevented the school from releasing a member of staff to chaperone the peer-supporters off site. The purpose of off-site delivery is to avoid in-school distractions (e.g., other pupils, prior commitments for example at lunch time, space constraints), to allow peer-supporters to work together and focus on a shared goal with their (non-school teacher) trainers, and to inspire peer-supporters with a special or “grown up” experience. As such, it would be ideal to deliver peer-supporter training off the school site. In future implementations of PLAN-A it would therefore be helpful to develop a persuasive pitch to school head teachers explaining the relatively low burden placed on school staff by PLAN-A, compared to other interventions [12, 21, 44, 45] to garner their support for off-site peer-supporter training. The intervention delivery, receipt and fidelity (including analysis of the school-based peer-supporter training) will be reported in full in a separate process evaluation paper.

We showed that the research required to evaluate the effectiveness and cost-effectiveness of the intervention in a larger trial is feasible. Questionnaire data provision was very high (>90%) at all time points. Accelerometer return rates exceeded >85% at each time point, however compliance with the wear time criteria decreased to 62% at Time 2 and was 5-7% lower in the control versus intervention group. Despite this, the level of completely missing accelerometer data (i.e., not wearing the monitor at all on a given day) was low. This suggests that whilst participants were willing to wear the accelerometers, strategies are needed to ensure that they are worn for long enough on measurement days, especially amongst control group participants, in a future definitive trial. Such strategies could include incentivising sufficient wear time rather than, or in addition to, monitor return [46, 47], amending the study design to reduce participant fatigue (i.e., accelerometery at baseline and beginning of Year 9 in the first instance), or using alternative accelerometers (e.g., wrist worn units) which may be less obtrusive and more comfortable. Even using these strategies, some missing accelerometer data is inevitable, and we also showed that imputation of missing accelerometer data was feasible and had no discernible impact on the estimates of intervention effectiveness.

The study showed that the PLAN-A intervention has the potential to positively affect adolescent girls’ MVPA (≈ +6 minutes per weekday) and reduce their sedentary time (≈ -23 minutes per weekday) compared to controls 4-5 months post-intervention. The estimates in this study suggest that the between-arm difference represents a stemming of the decline in MVPA that was seen in the control group. Previous school-based physical activity interventions for adolescent girls show very small effects [10] and some multicomponent school-based interventions were more effective for boys than girls [12]. The findings of the present study suggest that PLAN-A has the potential to have a larger effect than previous interventions and provides initial support for the alternative peer-led physical activity intervention model used in PLAN-A.

The evidence presented above suggests that the conduct of a larger definitive trial to determine the effectiveness and cost-effectiveness of the PLAN-A intervention is warranted. Sample size calculations suggested that this study should include 20 schools (ten intervention & ten control) and 1400 Year 8 girls to have sufficient statistical power to detect a 10-minute between-arms difference in MVPA. The details of minor amendments to the intervention content that were identified in the process evaluation will be described in a separate paper. In comparison to other studies PLAN-A has the potential to be good value. PLAN-A cost £37 per Year 8 girl and £6 per additional minute of weekday MVPA (based on the between-arms comparison at Time 2) which compares favourably, for example, to the multicomponent Physical Activity 4 Everyone trial [48] which had an intervention cost over 24 months of £240 per student and a difference in mean daily MVPA of 7.0 minutes (95% CI: 2.7 to 11.4) which equated to £34 (95% CI: £21 to £90) per additional minute of MVPA.

### Strengths and Limitations

A main strength of this study was its methodology. The PLAN-A intervention was carefully adapted from an effective adolescent smoking cessation intervention model through formative work with key stakeholders [40]. As such, the intervention used a novel means of peer-support for physical activity. The study was aligned with guidance on the development of complex interventions [49] and provided important testing of procedures, estimates of recruitment, indicators of intervention feasibility and promise to change behaviour and determination of the sample size required for a future effectiveness trial. Further strengths are that the study conduct adhered to the previously published study protocol [25], the use of an independent study oversight group, the use of accelerometers to measure physical activity, and the grounding of the intervention on complementary theories of peer-influence and motivation. A limitation of the study is the involvement of only two control schools which provides little information on recruiting and retaining schools in the control arm. Further, there was limited ethnic diversity of participants across all schools and additional research is needed to explore feasibility in schools with a more ethnically diverse pupil population. Finally, the relatively high level of missing accelerometer data at the second follow-up would need to be improved in a future trial.

## Conclusions

It is feasible to implement a secondary school-based peer-led physical activity intervention for adolescent girls based on trained influential students providing informal support to their year group peers. It is also feasible to study this intervention using a robust cluster-randomised trial design. The intervention shows promise to stem the age-related decline in adolescent girls’ physical activity, and overall the evidence presented suggest that a definitive trial is warranted to investigate the effectiveness and cost-effectiveness of the intervention.

## Additional files

Additional file 1: Consort 2010 extension for pilot and feasibility trials checklist. Completed CONSORT checklist (version adapted for reporting pilot and feasibility studies). (DOC 225 kb)

Additional file 2: Template for Intervention Description and Replication (TIDieR) checklist for PLAN-A.Completed TIDieR Checklist describing the PLAN-A intervention. (DOCX 16 kb)

Additional file 3: Baseline descriptive statistics of peer-supporter and non-peer-supporter students. Descriptive data for peer-supporters and non-peer-supporters at baseline. (DOCX 15 kb)

Additional file 4: EQ-5D-Y and QALYs results, by treatment arm. Baseline, Time 1 and Time 2 EQ-5D-Y results and corresponding QALY estimates, by treatment arm (DOCX 14 kb)

Additional file 5: Sensitivity analysis of evidence of promise for intervention effects on weekday MVPA at Time 1 and Time 2 using the imputed data set and complete data where accelerometer wear was one or more valid day. Sensitivity analysis of evidence of promise for intervention effects on weekday MVPA at Time 1 and Time 2 using the imputed data set and complete data where accelerometer wear was one or more valid day (DOCX 13 kb)

Additional file 6: Exploratory interaction of the intervention effect on weekday MVPA at Time 1 and Time 2 and peer supporter status. Exploratory interaction of the intervention effect on weekday MVPA at Time 1 and Time 2 and peer supporter status. (DOCX 13 kb)

## Abbreviations

DOI: Diffusion of innovations
ICC: Intra-class correlation
IMD: index of multiple deprivation
MVPA: moderate to vigorous physical activity
PLAN-A: Peer-Led physical Activity iNtervention for Adolescent girls.
SDT: Self-determination theory
