# Supplementary material for: Results of a feasibility cluster randomised controlled trial of a peer-led school-based intervention to increase the physical activity of adolescent girls (PLAN-A)
Source: Int J Behav Nutr Phys Act. 2018 Jun 7;15:50. doi: 10.1186/s12966-018-0682-4 (PMC5992776; doi:10.1186/s12966-018-0682-4)
Supplement: Supplementary file 2 — Template for Intervention Description and Replication (TIDieR) checklist for PLAN-A.Completed TIDieR Checklist describing the PLAN-A intervention. (DOCX 16 kb) [file 12966_2018_682_MOESM2_ESM.docx]

Additional file 2. Template for Intervention Description and Replication (TIDieR) checklist for PLAN-A

| **Item** | **Description** |
| --- | --- |
| Name (1) | Peer-Led physical Activity iNtervention for Adolescent girls (PLAN-A) |
| Why (2) | The primary aim of this study was to assess the feasibility of the PLAN-A peer-led intervention designed to increase the PA levels of adolescent girls. The aims of the feasibility study were to estimate recruitment, retention and attendance rates, examine the acceptability of the intervention to schools, trainers, Peer-supporters, Non-peer-supporter pupils, peer-supporter trainers and parents. The study also sought to test the feasibility of data collection and assess data provision rates as well as examine intervention effect on accelerometer derived MVPA. In addition, estimate school-related ICC for daily MVPA and sample size for a definitive trial.  The MVPA levels of girls are lower than boys throughout childhood and adolescents, declining at a steeper rate. As girls become older, it is thought that this decline can be contributed to not only changes in their social context (friendship groups/peer support), but the perception of significant barriers to physical activity that begin to emerge. Research suggests that there is an urgent need for effective physical activity interventions for girls.  Peers, through peer support, co-participation and creating positive peer norms for activity, can play a central role in adolescent girls’ physical activity. Social network research has also revealed that children within the same friendship group tend to have similar activity levels and may alter their activity levels to match their friends’.  Utilising ‘peer power’ in an intervention alone may not be enough to see positive changes in PA behaviour. PLAN-A is based on Diffusion of Innovations theory, the fundamental framework for using influential *change agents* such as peer-supporters. PLAN-A is also built on Self-Determination Theory (SDT) which aims to foster autonomous motivation for physical activity by satisfying autonomy, competence and social belonging amongst the girls. |
| What: Materials (3) | Training (train-the-trainers) was provided for the trainers hired to deliver the peer-supporter training. The training took place over the course of three consecutive days. Content covered the concept and principles behind PLAN-A, the theoretical underpinnings of the intervention and how to deliver the training to uphold these (particularly when in a challenging environment) and a rehearsal of all the activities that formed the peer-supporter training. Slides were used to support delivery.  Each trainer was provided with a ‘Trainers’ guide’ introducing PLAN-A (including its rationale and aims), detailing SDT principles, their role as a trainer, any practicalities or logistics, how to use the ‘Session plans’ and a run through of the train-the-trainer training days. The ‘PLAN-A Session plans’ consisted of a complete guide to all the activities to be delivered during the two-day and top-up day peer-supporter training. The manual gave exact details of each activity, as well as resources needed, how to prepare and objectives for each activity. Each trainer pair received a resource pack containing worksheets and materials to help deliver the training, as well as any technical equipment needed.  Each peer-supporter received a ‘peer-supporter booklet’ combined with a ‘Diary’. The booklet contained supporting materials and answers to various activities held within the training. The diary provided the peer-supporters with an opportunity to record and reflect on the conversations they may have had with peers. |
| What: Procedures (4) | Recruitment letters were sent to secondary schools in the South Gloucestershire and Wiltshire council areas. Once schools expressed interest, a meeting was held between the PLAN-A project manager and school contact for further discussion and to obtain a signed study agreement form. A student recruitment briefing was then held for all Year 8 girls in which they were told about PLAN-A and given information packs for themselves and their parents. Parents signed an opt-out form if they did not wish their daughter to take part.  Peer nomination and data collection were held in all schools. Schools were then randomly assigned to intervention (n = 4) or control (n = 2) groups and were made aware of their allocation post baseline data collection. Peer nomination analysis was completed for each intervention school.  A peer-supporter meeting was held with all the nominated peer-supporter from each intervention school. This covered details about the role of a peer-supporter and the logistics and content of the training. Peer-supporter were given information packs for themselves and their parents. And were asked to return a signed parent consent and student assent form in order to attend the training.  All peer-supporters received an initial two-day training, delivered by two trainers. Half way through the intervention period (approximately 5 weeks later) they received a top-up day training session. The training gave the peer-supporter the skills, knowledge and confidence to fulfil the role. Once training was complete, peer-supporter returned to school and diffused informal messages encouraging their peers to be active.  A process evaluation, using quantitative and qualitative methods, was conducted to identify areas of success and required improvement, the acceptability of the intervention and its design, as well as potential mechanisms of impact. Attendance at peer-supporter training was recorded by trainers, observations of each trainer pair on the two-day and top-up day training were carried out to assess intervention fidelity, logistics and peer-supporter engagement. Peer-supporters and trainers completed a post-two-day and top-up day training evaluation form. Peer-supporters reported on enjoyment, knowledge gained, concerns about being a peer-supporter and perceived trainer autonomy support. Trainers reported on fulfilment of objectives, logistics and perceived student engagement. Semi-structured interviews and focus groups were conducted with trainers, peer-supporters, parents of peer-supporters, school contacts and non- peer-supporters to gain feedback about the successes and any issues with the intervention. A school context audit was completed to assess level of physical activity provision, school policies to support physical activity and attitude towards physical activity. |
| Who provided (5) | Female trainers with a background in delivering physical activity programmes, working with young people or in theatre were recruited to the study to deliver the peer-supporter training. Five trainers delivered the intervention, one trainer was unable to deliver any top-up day training, and therefore another trainer took thier place. All trainers completed a three-day train-the-trainers programme approximately one week before delivery. Instructors were paid to attend the train-the-trainers and for each peer-supporter training they delivered. |
| How (6) | Peer-supporter training was provided for each school to the top ~18% of girls nominated to be a peer-supporter (range: 11 – 17) and delivered by two trainers. Peer-supporter then returned to school and informally diffused messages about PA to their friends. |
| Where (7) | Peer-supporter training was delivered during the school day. Schools were located in South Gloucestershire and Wiltshire. The two-day and top-up day sessions were delivered off school site, usually in a town / local hall. Peer-supporter training for one school was held within school grounds because the school were unable to release a staff member to attend the training. |
| When and how much (8) | Intervention schools received the initial two-day peer-supporter training in February 2016 and received the top-up day mid-way through the intervention period in April 2016. The training days ran from ~9.00 – 15.00 to reflect the usual school day. The intervention was ten weeks in duration for the purposes of defining the first follow up point, however peer-supporter were encouraged to peer support for as long as they felt necessary, therefore there was formal no defined period. |
| Tailoring (9) | All trainers received the same training and resources for delivery and were encouraged to deliver the peer-supporter training in an autonomy-supportive style consistent with SDT. Peer-supporter from all schools received the same training, but delivered by different trainers, sessions were observed for intervention fidelity. |
| Modifications (10) | No modifications were made to the intervention. |
| How well: Planned (11) | Observations assessed intervention fidelity, including the extent to which the trainers met activity objectives, delivered in an autonomy supportive style and activities that were missed. Peer-supporter, non-peer-supporter and trainer focus groups and interviews evaluated the extent to which peer-supporter had provided support and what impact this may have had. |
| How well: Actual (12) | Across all three training days, fulfilment of activity objectives was rated as 2.66 ± 0.58 (Mean ± SD) out of 3, suggesting that the majority of training objectives were achieved. Observations witnessed that for the majority of the training, trainers adhered to the principles of SDT by ensuring peer-supporters’ opinions felt valued, attempting to provide choice and making efforts to bond with the girls. Activities that were not delivered were those that were suggested in the manual to be missed if trainers needed to make up time.  Both peer-supporters and trainers reported making efforts to support and encourage peers to be more active by providing knowledge, co-participating in physical activity, and being subtle in their attempts. Some non-peer-supporter were sceptical about the amount of peer support they had received although peer-supporters believed that this was partly based on the informal, subtle (rather than formal and overt) support they gave. |
