# Supplementary material for: Results of a feasibility cluster randomised controlled trial of a peer-led school-based intervention to increase the physical activity of adolescent girls (PLAN-A)
Source: Int J Behav Nutr Phys Act. 2018 Jun 7;15:50. doi: 10.1186/s12966-018-0682-4 (PMC5992776; doi:10.1186/s12966-018-0682-4)
Supplement: Supplementary file 3 — Baseline descriptive statistics of peer-supporter and non-peer-supporter students. Descriptive data for peer-supporters and non-peer-supporters at baseline. (DOCX 15 kb) [file 12966_2018_682_MOESM3_ESM.docx]

**Additional file 3**: Baseline descriptive statistics of peer-supporter and non-peer-supporter students

|  | **Peer-supporters**^a^ | | **Non-peer-supporters** | |
| --- | --- | --- | --- | --- |
| **Variable** | **n** | **Mean ± SD / Median (LQ, UQ) / %** | **n** | **Mean ± SD / Median (LQ, UQ) / %** |
| Age | 83 | 13.59 ± 0.27 | 344 | 13.50 ± 0.30 |
| IMD | 78 | 10.99 (5.69, 18.60) | 301 | 11.05 (6.77, 18.20) |
| Family affluence | 83 | 7.48 ± 1.48 | 343 | 6.82 ± 1.64 |
| Receiving free school meals (n, %) | 5 | 6.02 | 47 | 13.70 |
| Ethnicity – White British (n, %) | 75 | 90.36 | 282 | 82.22 |
| Ethnicity – White other (n, %) | 1 | 1.20 | 17 | 4.96 |
| Ethnicity – Mixed (n, %) | 2 | 2.41 | 10 | 2.92 |
| Ethnicity – Other (n, %) | 5 | 6.02 | 34 | 9.91 |
| Weekday MVPA (min) | 71 | 63.83 (51.43, 77.88) | 282 | 51.79 (40.67, 67.92) |
| Weekend MVPA (min) | 41 | 49.83 (35.67, 68.00) | 183 | 37.17 (21.92, 56.83) |
| Overall MVPA (min) | 71 | 60.36 (50.50, 72.56) | 282 | 49.79 (39.69, 64.83) |
| Weekday sedentary (min) | 71 | 469.71 (422.67, 524.37) | 282 | 498.63 (437.27, 546.47) |
| Weekend sedentary (min) | 40 | 428.71 (377.17, 469.08) | 183 | 437.83 (385.08, 526.50) |
| Overall sedentary (min) | 71 | 418.03 (60.57, 489.89) | 282 | 448.90 (67.11, 518.33) |
| 60 mins MVPA per weekday (n, %) | 42 | 59.15 | 99 | 35.11 |
| 60 mins MVPA per weekend day (n, %) | 13 | 31.71 | 43 | 23.50 |
| EQ-5D-Y | 82 | 80.21±14.19 | 331 | 73.24 ± 19.05 |

IMD = Index of Multiple Deprivation; MVPA = moderate-to-vigorous physical activity. ^a^ Peer-supporters are those nominated in both intervention and control schools
