# Supplementary material for: Results of a feasibility cluster randomised controlled trial of a peer-led school-based intervention to increase the physical activity of adolescent girls (PLAN-A)
Source: Int J Behav Nutr Phys Act. 2018 Jun 7;15:50. doi: 10.1186/s12966-018-0682-4 (PMC5992776; doi:10.1186/s12966-018-0682-4)
Supplement: Supplementary file 4 — EQ-5D-Y and QALYs results, by treatment arm. Baseline, Time 1 and Time 2 EQ-5D-Y results and corresponding QALY estimates, by treatment arm (DOCX 14 kb) [file 12966_2018_682_MOESM4_ESM.docx]

**Additional File 4**: EQ-5D-Y and QALYs results, by treatment arm

|  | **Intervention** | | | **Control** | | | **Difference in means (95% CI)** |
| --- | --- | --- | --- | --- | --- | --- | --- |
|  | **n** | **Mean** | **(SD [95% CI])** | **n** | **Mean** | **(SD [95% CI])** |  |
| Time 0 | 264 | 0.837 | (0.202 [0.813, 0.861]) | 152 | 0.844 | (0.208 [0.811, 0.878]) | -0.007 (-0.048, 0.033) |
| Time 1 | 254 | 0.812 | (0.227 [0.784, 0.840]) | 143 | 0.836 | (0.185 [0.805, 0.866]) | -0.024 (-0.067, 0.020) |
| Time 2 | 249 | 0.831 | (0.204 [0.806, 0.857]) | 141 | 0.875 | (0.176 [0.846, 0.904]) | -0.044 (-0.084, -0.004) |
| QALYs^a^ | 238 | 0.830 | (0.176 [0.808, 0.852]) | 130 | 0.856 | (0.142 [0.831, 0.880]) | -0.015 (-0.038 to 0.007) |

^a^ Difference in means adjusted for baseline EQ-5D-Y, trial arm, LEA and school level clusters
