# Supplementary material for: Results of a feasibility cluster randomised controlled trial of a peer-led school-based intervention to increase the physical activity of adolescent girls (PLAN-A)
Source: Int J Behav Nutr Phys Act. 2018 Jun 7;15:50. doi: 10.1186/s12966-018-0682-4 (PMC5992776; doi:10.1186/s12966-018-0682-4)
Supplement: Supplementary file 5 — Sensitivity analysis of evidence of promise for intervention effects on weekday MVPA at Time 1 and Time 2 using the imputed data set and complete data where accelerometer wear was one or more valid day. Sensitivity analysis of evidence of promise for intervention effects on weekday MVPA at Time 1 and Time 2 using the imputed data set and complete data where accelerometer wear was one or more valid day (DOCX 13 kb) [file 12966_2018_682_MOESM5_ESM.docx]

**Additional file 5**.

Sensitivity analysis of evidence of promise for intervention effects on weekday MVPA at Time 1 and Time 2 using the imputed data set and complete data where accelerometer wear was one or more valid day.

|  | **Control** | | **Intervention** | |  |
| --- | --- | --- | --- | --- | --- |
|  | **n** | **Mean ± SD** | **n** | **Mean ± SD** | **Intervention vs. Control**  **adjusted difference in means (95% CI) ^a^** |
|  | **Imputed data** | | | | |
|  | **Time 1** | | | | |
| MVPA per weekday (mins)^b^ | 158 | 61.72 **±** 22.84 | 269 | 60.66 **±** 24.19 | 2.70 [-2.30, 7.71] |
|  | **Time 2** | | | | |
| MVPA per weekday (mins)^b^ | 158 | 55.25 **±** 20.34 | 269 | 59.69 **±** 23.19 | 6.89 [2.60, 11.18] |
|  | **Complete data where ≥ 1 valid day of accelerometer data was provided** | | | | |
|  | **Time 1** | | | | |
| MVPA per weekday (mins)^b^ | 110 | 62.45 **±** 22.87 | 214 | 62.17 **±** 26.19 | 2.42 [-2.60, 7.44] |
|  | **Time 2** | | | | |
| MVPA per weekday (mins)^b^ | 102 | 55.00 **±** 20.86 | 193 | 58.90 **±** 22.84 | 6.29 [1.69, 10.90] |

^a^ The control group is the reference group for between group comparisons. Models are adjusted for baseline outcome value, N valid days accelerometer data, local authority and school-level clustering

^b^ *A priori* primary outcome in a future definitive trial
