# Supplementary material for: Results of a feasibility cluster randomised controlled trial of a peer-led school-based intervention to increase the physical activity of adolescent girls (PLAN-A)
Source: Int J Behav Nutr Phys Act. 2018 Jun 7;15:50. doi: 10.1186/s12966-018-0682-4 (PMC5992776; doi:10.1186/s12966-018-0682-4)
Supplement: Supplementary file 6 — Exploratory interaction of the intervention effect on weekday MVPA at Time 1 and Time 2 and peer supporter status. Exploratory interaction of the intervention effect on weekday MVPA at Time 1 and Time 2 and peer supporter status. (DOCX 13 kb) [file 12966_2018_682_MOESM6_ESM.docx]

**Additional file 6.** Exploratory interaction of the intervention effect on weekday MVPA at Time 1 and Time 2 and peer supporter status.

|  | **Control** | | **Intervention** | | **Intervention vs. control adjusted difference in means (95% CI)^a^** |
| --- | --- | --- | --- | --- | --- |
|  | **n** | **Mean ± SD** | **n** | **Mean ± SD** |  |
|  | **Time 1** | | | | |
| Whole sample | 95 | 61.19 **±** 22.10 | 177 | 60.72 **±** 22.45 | 1.11 [-4.31, 6.55] |
| Peer-supporters^b^ | 16 | 57.49 **±** 27.34 | 40 | 65.97 **±** 20.64 | 14.20 [3.16, 25.25] |
| Non-peer-supporters | 79 | 61.94 **±** 21.01 | 137 | 59.19 **±** 22.80 | -0.85 [-5.53, 3.83] |
| Interaction between Cohort and trial arm on weekday MVPA | | | | | p=0.018 |
|  | **Time 2** | | | | |
| Whole sample | 81 | 54.31 **±**19.56 | 162 | 58.65 **±** 22.21 | 6.09 [1.43, 10.76] |
| Peer-supporters | 12 | 60.14 **±** 15.15 | 40 | 61.73 **±** 22.47 | 2.06 [-9.35, 13.48] |
| Non-peer-supporters | 69 | 53.30 **±** 20.15 | 122 | 57.65 **±** 22.12 | 6.69 [1.63, 11.76] |
| Interaction between Cohort and trial arm on weekday MVPA | | | | | p=0.78 |

^a^ Models are adjusted for baseline outcome value, N valid days accelerometer data & local authority. Analysis of complete cases. ^b^ Peer-supporter nomination was carried out in all schools, allowing the identification of pupils who would have been invited to be peer-supporters in control schools.
